# Voltage control of magnetism in Ni–Co oxide mesoporous films: impact of porosity on oxygen magneto-ionics performance

**DOI:** 10.1039/d6nr00524a

**Published:** 2026-04-22

**Authors:** Aitor Arredondo-López, Konrad Eiler, Alberto Quintana, Zheng Ma, Maciej Oskar Liedke, Eric Hirschmann, Andreas Wagner, Enric Menéndez, Jordi Sort, Eva Pellicer

**Affiliations:** a Departament de Física, Universitat Autònoma de Barcelona E-08193 Cerdanyola del Vallès Spain enric.menendez@uab.cat jordi.sort@uab.cat eva.pellicer@uab.cat; b Catalan Institute of Nanoscience and Nanotechnology (ICN2), CSIC and BIST 08193 Barcelona Spain; c Institute of Radiation Physics, Helmholtz-Zentrum Dresden – Rossendorf Dresden 01328 Germany; d Institució Catalana de Recerca i Estudis Avançats (ICREA) Pg. Lluís Companys 23 E-08010 Barcelona Spain

## Abstract

Control of magnetism through electric-field-driven migration of ions, referred to as magneto-ionics (MI), holds promise for the development of non-volatile energy-efficient memory storage, as well as spintronic, neuromorphic and magnetoelectric devices. Here, we study the MI phenomena in 350 nm thick Ni_55_Co_45_ oxide films with varying degrees of porosity, obtained by electrodeposition of the parent Ni–Co metallic alloy on metallized Si substrate and subsequent annealing in air. Annealing at 450 °C of the film electrodeposited from a P-123-containing electrolyte with Ni and Co sulfate salts yields a Ni–Co oxide that partially retains its mesoporosity. This sample exhibits a higher MI response compared to a low-porosity (nearly dense) Ni–Co oxide film, indicating that an increased surface-to-volume ratio enhances MI. Comprehensive characterization of the mesoporous Ni–Co oxide-coated Si/Ti/Au sample reveals that annealing not only oxidizes the top ≈100 nm of the Ni–Co film but also induces silicon diffusion. MI phenomena occur *via* O^2−^ migration out of and into the top Ni–Co oxide layer under negative and positive biasing, respectively. While the system shows some irreversibility, endurance improves significantly as cycling frequency increases, evidencing the potential of this material for voltage-tunable memory applications.

## Abstract 2

Nanoporosity in Co–Ni oxide films grown by electrodeposition and air-annealing improves oxygen magneto-ionics and cyclability compared to dense counterparts.

## Graphical abstract

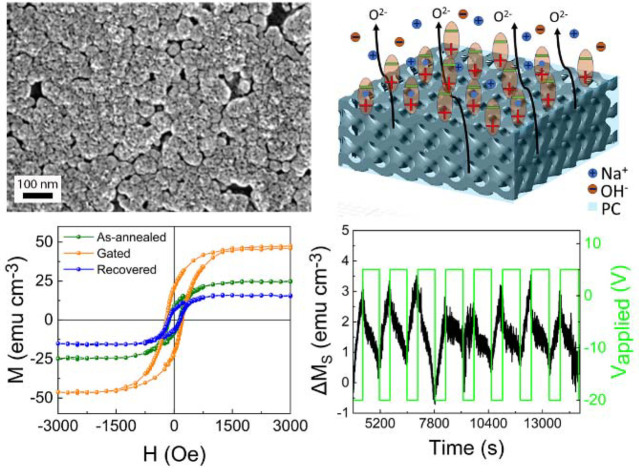

## Introduction

The advent of Big Data has created a growing demand for innovative, energy-efficient data processing methods.^1,2^ Traditionally, the manipulation of magnetic bit orientation has relied heavily on electric currents, with advancements such as miniaturized electromagnets and devices that operate with spin-polarized electric currents (*e.g.*, spin-transfer-torque magnetoresistive random access memories (STT-MRAMs)) developed for this purpose. In STT-MRAMs, power dissipation in the form of Joule heating^3^ continues to be a significant challenge, particularly as devices shrink in size.^4,5^ The use of electric fields (*i.e.*, voltage) instead of electric currents to control magnetism through the converse magnetoelectric (CME) effect^3,6^ in miniaturized systems could provide a more energy-efficient solution for magnetic data storage and computing devices. This becomes particularly relevant in the context of emerging research fields such as neuromorphic computing, where voltage pulses are used to mimic the way the human brain processes information.^7–9^ Despite ongoing intensive research on CME, the search for new materials to improve the performance of data storage technologies remains critical.

In recent decades, the manipulation of the magnetic state of materials using electric fields has been extensively demonstrated. Notable examples include: (i) single-phase multiferroics,^10^ (ii) ferroelectric/magnetostrictive composites,^11^ and (iii) ultrathin metallic films.^12^ However, each of these materials presents challenges: (i) most complex multiferroic oxides operate mainly at low temperatures, (ii) fatigue effects limit endurance of multiferroic heterostructures, and (iii) the Thomas–Fermi screening length (typically 0.5–1 nm)^13^ in metals restricts effective magnetoelectric effects to the surface, respectively.

Magneto-ionics (MI), which involves changes in the magnetic properties of materials arising from electric-field-driven migration of ions (H^+^, N^3−^, O^2−^, F^−^, OH^−^),^14,15^ offers a compelling alternative to conventional CME mechanisms. In simple terms, applying a voltage induces ion diffusion within the material – commonly referred to as the MI target. MI can operate in solid state or in liquid form (*i.e.*, liquid electrolyte gating).^15^ For the latter, the MI target (also serving as the working electrode) and the counter electrode are immersed in a suitable liquid (*e.g.*, polar aprotic electrolytes or ionic liquids). When voltage is applied, voltage-induced ionic transport can significantly modify magnetic properties such as saturation magnetization, coercivity, Curie temperature, exchange bias, Ruderman–Kittel–Kasuya–Yosida (RKKY) interactions, the anisotropy easy axis, or skyrmions density, among others.^14–18^ Key advantages of MI include non-volatile effects and compatibility with a wide range of film thicknesses.

An important advantage of MI actuation *via* liquid electrolyte gating is that charges can accumulate effectively at the material/liquid interface, leading to the formation of an electric double layer (EDL), with a thickness ranging from 0.5 nm to 1 nm. The strength of the electric field relies on the efficient formation and restructuring of the EDL when either positive or negative bias is applied. One way to enhance MI effects is by introducing controlled porosity into the magneto-ionic material, thereby increasing the material–liquid interface area.

Mesoporous materials have large surface areas due to their pores, which range in size from 2 nm to 50 nm.^19–21^ As a result, they find uses in applications where surface is a key property such as photo- and electrocatalysis, gas sensing or energy storage devices (*e.g.*, electrodes in batteries and supercapacitors).^22^ Several strategies have been put forward to engineer mesoporous metals and metal oxides in thin film form.^23^ In the field of CME, significant modulation of coercivity (*H*_C_) has been shown in mesoporous metallic films like Fe–Cu^24^ or Cu–Ni.^25^ Specifically, nanoporous Cu–Ni showed up to a 32% reduction in *H*_C_ upon negative voltage application,^25^ a six-fold greater change compared to non-porous FePt and FePd ultrathin dense films, where coercivity changes were around 4.5%.^13^ The modulation in *H*_C_ was attributed to changes in the magnetic anisotropy energy. The reduction in *H*_C_ in these ferromagnetic materials allows for magnetization reversal at much lower applied magnetic fields, thereby minimizing energy consumption during the writing process of magnetic information. Less attention has been given to the potential for modulating the saturation magnetization (*M*_S_) of mesoporous films *via* voltage-induced ion migration.

Aqueous electrodeposition from surfactant assemblies offers a highly effective approach for creating mesoporous metallic films on a conductive substrate. The underlying principle of this method involves adding surfactants to the electrolyte just above their critical micelle concentration. Under these conditions, metal cations coordinate with the hydrophilic shell of the micelles, directing the formation of a mesoporous network on the substrate. Mesoporous metal films have been successfully electrodeposited using non-ionic surfactants such as Brij-56, Brij-58, F-127, and P-123.^26,27^ This method has proven successful for fabricating mesoporous films of single metals, while creating binary alloys – especially those containing both noble and non-noble metals – remains more challenging due to the differing electrochemical reduction potentials of the metal salts in solution.^28^

In this study, we observe a porosity-dependent, voltage-driven magnetic state switching in mesoporous Ni–Co oxide films. Metallic Ni–Co films, approximately 350 nm in thickness, were obtained by electrodeposition on metallized silicon substrates and subsequently annealed in air to achieve a Ni–Co oxide with relatively low magnetization that enables O^2−^ MI. Galvanostatic electrodeposition was performed using both P-123-containing and P-123-free aqueous electrolytes to produce mesoporous and dense Ni_55_Co_45_ films, respectively. The annealing temperature was carefully selected to oxidize the as-deposited mesoporous metallic films while maintaining their mesoporosity (*i.e.*, preventing the complete collapse of the mesopores). Liquid electrolyte gating of the resulting Ni–Co oxide films was carried out in propylene carbonate (PC) to induce oxygen ion migration across the material towards the liquid electrolyte, which also serves as oxygen ion reservoir. For the mesoporous Ni–Co oxide film exhibiting a higher modulation of its magnetic moment at saturation with voltage, a comprehensive characterization using variable energy positron annihilation lifetime spectroscopy (VEPALS), X-ray diffraction (XRD), X-ray photoelectron spectroscopy (XPS), and transmission electron microscopy (TEM) was performed to correlate the changes in magnetic properties with structural and chemical modifications. Our results reveal that the presence of porosity was advantageous for MI.

## Experimental

### Substrate metallization

A Ti (10 nm)/Au (90 nm) buffer/seed layer was sputtered onto a Si wafer using an AJA International Inc. Sputtering system at room temperature. The base pressure was 1.7 × 10^−7^ bar while the working pressure during sputtering was set at 3 mTorr. The Ti layer was deposited from a Ti target at 200 W (DC) for 10 s, after which a layer of Au was sputtered at 100 W (DC) for 1 min. The metallized Si wafers were sliced into chips of 0.5 cm × 1 cm.

### Growth of mesoporous Ni–Co thin films

The electrodeposition of Ni–Co films was carried out in a three-electrode cell using a PGSTAT204 Autolab potentiostat/galvanostat controlled with NOVA 2.0 software. The previously sliced Si/Ti/Au chips were used as cathodes (0.25 cm^2^ exposed area). A Pt spiral served as counter electrode and a double junction Ag/AgCl (*E*° = +0.210 V per SHE) with 3M KCl as inner solution and 1 M Na_2_SO_4_ as outer solution was used as the reference electrode (Metrohm AG). Prior to electrodeposition, the metallized silicon substrates were degreased with acetone (99% purity) and isopropanol (99.9% purity), followed by rinsing with Milli-Q water. Three different baths were used, whose formulations are shown in Table 1. Ni and Co salts (98% purity), H_3_BO_3_ (99.5% purity), and NH_4_Cl (99.99% purity) were purchased from Merck. Two of the baths contained P-123 (HO(CH_2_CH_2_O)_20_(CH_2_CH(CH_3_)O)_70_(CH_2_CH_2_O)_20_H) to promote mesoporosity, whereas P-123 was excluded from Bath 3 since this was intended to produce a low-porosity (nearly dense) coating. In all cases, the pH was adjusted to 2.3 with sulfuric acid. The Ni–Co films were deposited galvanostatically at −40 mA cm^−2^ and 25 °C, under stirred conditions (Table 2). The deposition time and stirring rate were adjusted for each bath to obtain Ni–Co films of comparable thickness.

**Table 1:** Chemicals and their concentrations for the three electrolytes used in the electrodeposition of Ni–Co films on Si/Ti/Au substrates. Note that Baths 1 and 2 contained P-123 as a structure-directing agent to produce mesoporous Ni–Co films while Bath 3 did not include P-123, resulting in dense Ni–Co films

Bath	Concentration (mM)							
	Ni_2_SO_4_·7H_2_O	NiCl_2_	Co_2_SO_4_·7H_2_O	C_2_H_5_NO_2_	H_3_BO_3_	NH_4_Cl	C_7_H_5_NO_3_S	P-123
1	150	—	8	175	150	50	—	10 g L^−1^
2	—	150	7.5	175	—	—	10	10 g L^−1^
3	150	—	8	175	150	50	—	—

**Table 2:** Electrodeposition parameters applied to produce the Ni–Co films from the three baths

Parameters	Bath		
	1	2	3
*j* (mA cm^−2^)	40	40	40
Time (s)	60	170	30
Stirring rate (rpm)	300	100	100
Bath temperature (°C)	25	25	25
pH	2.3	2.3	2.3

Once removed from the electrodeposition cell, the resulting Ni–Co films were cleaned by ultrasonic treatment in ethanol for 10 min to remove the P-123 from the metallic structure. This was followed by rinsing with Milli-Q water and an additional 30 min cleaning step using a UV cleaner (70 W, 254 nm).

### Annealing in air of the Si/Ti/Au/Ni–Co samples

The Si/Ti/Au/Ni–Co samples were subjected to annealing in air in a CARBOLITE tubular furnace at 450 °C. The samples were heated from room temperature to 450 °C at a rate of 3 °C min^−1^, held at 450 °C during 45 min and left to naturally cool down in air. This process aimed to oxidize the Ni–Co layer while preserving its mesoporosity, although complete oxidation could not be achieved since higher temperatures were found to result in complete collapse of the mesopores, making the chosen temperature a balanced compromise.

### Magnetoelectric characterization

Room-temperature hysteresis loops were acquired in a LOT-QuantumDesign MicroSense Vibrating Sample Magnetometer (VSM). The *in situ* liquid-gated magnetoelectric characterization was carried out in in-plane configuration (*i.e.*, applying the magnetic field along the plane of the samples) at room temperature, using an external power supply (Agilent B2902A). Voltages of −20 V and +5 V were applied consecutively between the Au seed layer of the samples and a Pt wire counter electrode. Upon electrical contact, the two electrodes were mounted into an Eppendorf which was filled with propylene carbonate (PC). The used electrolyte, PC, was previously treated with metallic Na to remove traces of water, causing the formation of Na^+^ and OH^−^ ions that contribute to enhance the ionic strength of the EDL.

### Defect characterization

Defect characterization was carried out by variable energy positron annihilation lifetime spectroscopy (VEPALS). VEPALS measurements were conducted at the Mono-energetic Positron Source (MePS) beamline at Helmholtz-Zentrum Dresden – Rossendorf (Germany).^29^ A CeBr_3_ scintillator detector together with a Hamamatsu R13089-100 photomultiplier tube for the gamma photon detection was employed. A Teledyne SPDevices ADQ14DC-2X digitizer with a 14-bit vertical resolution and 2 GS s^−1^ (gigasamples per second) horizontal resolution was utilized for the processing of signals.^30^ The overall time resolution of the measurement system is ≈0.250 ns and all spectra contain at least 1 × 10^7^ counts. A typical lifetime spectrum *N*(*t*), which is the absolute value of the time derivative of the positron decay spectrum, is described by:1
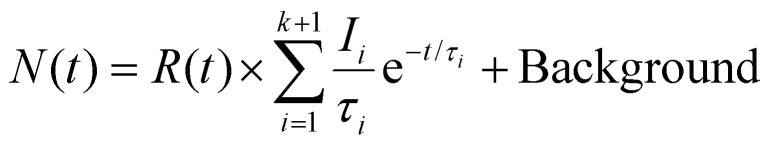
where *k* is the number of different defect types contributing to the positron trapping, which are related to *k* + 1 components in the spectra with individual lifetimes *τ*_*i*_ and relative intensities *I*_*i*_ (∑*I*_*i*_ = 1). The instrument resolution function *R*(*t*) is a sum of two Gaussian functions with distinct intensities and relative shifts both depending on the positron implantation energy, *E*_P_. *R*(*t*) was determined by measuring a reference sample, *i.e.* yttria-stabilized zirconia, which exhibits a known single lifetime component of 182 ± 3 ps. All the spectra were deconvoluted using a non-linear least-squares fitting method, minimized by the Levenberg–Marquardt algorithm in the software package PALSfit,^31^ into 3 major lifetime components, which directly evidence localized annihilation at 3 different defect types (sizes; *τ*_1_, *τ*_2_ and *τ*_3_). The shortest lifetime component *τ*_1_ represents smaller vacancy clusters, while the lifetime component *τ*_2_ accounts for larger vacancy clusters linked to grain boundaries or small pores. The longest lifetime component *τ*_3_ represents voids with diameters larger than 0.35 nm. The relative intensity (*I*_*i*_) of each component can be regarded to some extent as the concentration of each defect type. The positron lifetime and its intensity have been probed as a function of positron implantation energy *E*_*P*_, which is related to the mean implantation depth 〈*z*〉 following eqn (2):^32^2
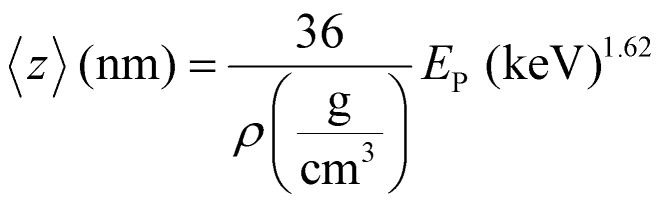


Note that 〈*z*〉 is an approximate measurement of depth since it does not account for positron diffusion.

### Topographical, compositional & structural characterization

A field-emission scanning electron microscope (FE-SEM, Zeiss Merlin) equipped with an energy dispersive X-ray (EDX) detector was used to characterize the morphology of the as-deposited and as-annealed Ni–Co-based films and their chemical composition. On-top SEM images were acquired at 1 keV in the in-Lens mode, whereas EDX analyses were done at 15 keV.

Sample thickness determination was carried out on a KLA P-15 Tencor mechanical profilometer. A 2 µm diamond stylus scanned the surface at a scan speed of 100 µm s^−1^, starting from a few microns corresponding to the Au seed-layer as a reference before stepping onto the Ni–Co oxide films. The applied force was set to 2 mg and the vertical range resolution was 0.0039 Å.

X-ray diffraction (XRD) patterns were carried out in a PANalytical X'pert Pro MRD diffractometer under *θ*/2*θ* geometry with the Bragg–Brentano configuration, using Cu K_α_ radiation (wavelength: *λ* = 0.15418 nm) and a 1D D/Tex detector. The recorded angular range spans from 35° to 60°.

X-ray photoelectron spectroscopy (XPS) experiments were performed on an ESFOSCAN equipment, which is based on a PHI 5000 VersaProbe instrument from Physical Electronics (ULVAC-PHI). Measurements were done with a monochromatic X-ray source (aluminum K_α_ line of 1486.6 eV) calibrated using the 3d^5/2^ line of Ag with a full width at half maximum of 0.6 eV. The analyzed area was a circle of 100 mm of diameter, and the selected resolution for the spectra was 224 eV of pass energy and 0.8 eV per step for the general spectra and 27 eV of pass energy and 0.1 eV per step for the high-resolution spectra of the selected elements. All measurements were made in an ultra-high vacuum (UHV) chamber at a pressure between 5 × 10^−10^ and 5 × 10^−9^ Torr. Prior to analysis, the utmost surface of the Ni–Co oxide films was sputtered with Ar ions for 4 min to remove contaminants. With the aim of collecting the Co 2p and Ni 2p core-level XPS spectra, the selected binding energy scan range was selected to be between 775 eV and 808 eV and between 850 eV and 889 eV, respectively. The spectra were corrected considering the position of carbon C 1s peak at 284.5 eV. The raw XPS data was treated with CasaXPS software, where a Shirley background was set as initial background conditions for all the peak fittings.

Transmission electron microscopy (TEM) observations were carried out on cross-sectional lamellae of Si/Ti/Au/Ni–Co oxide samples. A FIB Helios 5 UX, designed by ThermoFisher, was used to slice a rectangular cross-section of approximately 1 μm × 10 μm in top-view area. The preparation process involved the steps of successive coarse, medium and fine polishing at 30 kV and current ranges in between 90 and 26 pA, until reaching a thickness of 200 nm. As a last step, a low-energy fine thinning, aimed at achieving a final thickness of about 90 nm which made the lamella transparent, was done at 8 kV and 26 pA ion gun conditions. The last step involved a cleaning process at 5 kV for 20 s. TEM observations were performed on a Thermo Fisher Scientific Spectra 300 (S)TEM double-corrected TEM operated at 200 kV.

## Results and discussion

### Synthesis of Ni–Co oxide films and initial evaluation of their MI behavior

Three different baths were used for the growth of metallic Ni–Co films with varying porosity (Table 1). While Bath 3 was supposed to yield dense films, P-123 was added to Baths 1 and 2 to induce the development of porosity. Either bath contained Ni(ii) and Co(ii) salts as main electroactive species and glycine (C_2_H_5_NO_2_) as complexing agent.^33^ Boric acid served as a pH buffer, whilst ammonium chloride and saccharine (C_7_H_5_NO_3_S) acted as levelers.^34^ The addition of ammonium chloride and saccharin promoted the formation of smooth surfaces, making it easier to observe mesopores that might otherwise be obscured by pronounced grain morphology. The potential *versus* time curves recorded during the deposition of the Ni–Co films are shown in Fig. S1a. In all cases, a comparable thickness was measured by profilometry, of around 350 nm (see Fig. S1b). The amount of Ni in all films was 55 ± 3 at%, as confirmed by EDX (see a representative EDX spectrum in Fig. S2).

**Fig. 1:**
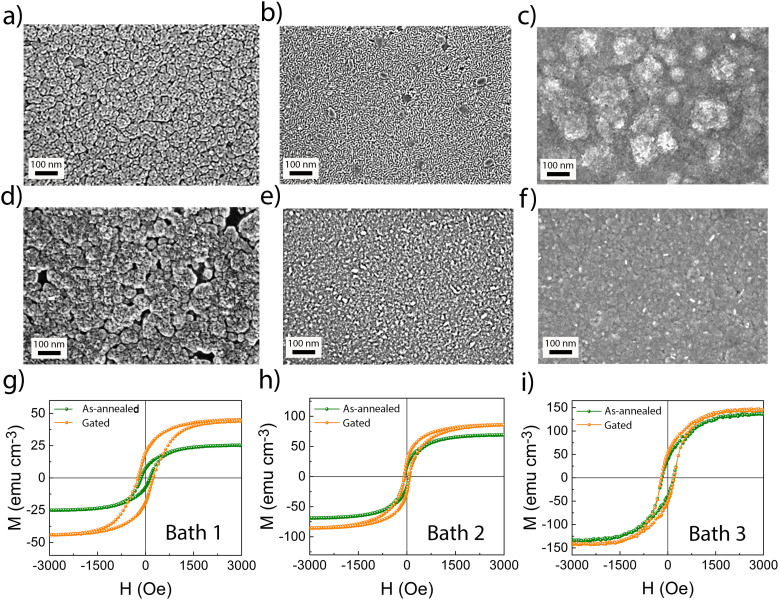
Top-view SEM images of the Ni–Co films obtained by electrodeposition from (a) Bath 1, (b) Bath 2, and (c) Bath 3. (d–f) are the corresponding top-view SEM images after annealing of the films at 450 °C for 45 min. (g–i) depict the room-temperature hysteresis loops of the Si/Ti/Au/Ni–Co oxide samples in the as-annealed state (green curves) and after gating at −20 V for 30 min (orange curves).

Fig. 1a–c show the top-view SEM images of the Ni–Co films obtained from Baths 1, 2, and 3, respectively. The Ni–Co film deposited from Bath 1 is composed of irregular grains with numerous tiny, spherical pores in the mesoporous size range (Fig. 1a), while the film derived from Bath 2 exhibits a worm-like mesoporosity (Fig. 1b). The formation of this type of porosity can be associated with the nature of the nickel salt. Compared to SO_4_^2−^, Cl^−^ is a weakly coordinating monovalent anion; therefore, Ni^2+^ ions can more readily approach the polyethylene oxide (PEO) chains of the P-123 micelles and coordinate with ether oxygen atoms. This interaction likely facilitates the development of a well-defined worm-like mesostructure. Contrarily, pores were not visible in the film obtained from Bath 3, as expected since it did not contain P-123 block copolymer. Hence, this film can be regarded as nearly ‘dense’. Note that Bath 3 contained the same chemicals as Bath 1 except for P-123 surfactant.

Annealing at 450 °C for 45 min in air was performed to oxidize the films while preserving their porosity in those obtained from Baths 1 and 2. Fig. 1d shows that mesoporosity was well preserved after annealing of the film electrodeposited from Bath 1, whereas the worm-like features in the film from Bath 2 largely disappeared (Fig. 1e), although a few voids remained. Annealing also caused morphological changes in the film obtained from Bath 3, but it remained dense as expected (Fig. 1f). The magnetization (M) *versus* applied magnetic field (*H*) measurements showed that the Ni–Co layer was not fully oxidized, as clear hysteresis loops were still observed (see the green curves in Fig. 1g–i). Based on the measured saturation magnetization (*M*_S_) of 25 emu cm^−3^ for this sample, and using a rough estimation that accounts for the stoichiometry of the Ni–Co oxide film, the *M*_S_ values of pure Co and Ni (161.8 emu g^−1^ and 55.1 emu g^−1^, respectively), their bulk densities (8.8 g cm^−3^ and 8.9 g cm^−3^, respectively), and the rule of mixtures, the result suggests that approximately 2.6% of the sample corresponds to a metallic Ni_55_Co_45_ alloy. Higher temperatures and longer annealing times could be applied to obtain a paramagnetic (‘OFF’) state or, at least, to further decrease M^35^ but they caused significant pore collapse, even in films synthesized from Bath 1, which greatly reduced their surface area (Fig. S3). Thus, there was a trade-off between attempting to achieve a fully paramagnetic response and preserving the porous character of the films. The Si/Ti/Au/Ni–Co oxide samples were liquid-gated at −20 V for 30 min, and the resulting magnetic changes were monitored *in situ* using VSM. Voltage application was managed through the use of PC, a polar aprotic liquid electrolyte with solvated Na^+^ (around 30 ppm) and OH^−^ ions, which is broadly utilized in different magnetoelectric systems.^24,35–37^ An increase in the magnetization (*M*) was observed in all three cases, with the relative change being significantly higher for the film derived from Bath 1. Specifically, the relative change in the magnetic moment at saturation was 8% for the dense film (Bath 3) and 25% for the film from Bath 2. In contrast, the film from Bath 1, which retained its mesoporosity after annealing, showed an 84% increase in the magnetic moment at saturation. This can be largely attributed to the increased surface area exposed to the electrolyte in this film, as observed in previous reports for metallic materials.^24,25,36^ We prove here that the same is true for mesoporous metal oxides. The evolution of *M vs*. time for the three Ni–Co oxide films is shown in Fig. S4 of the SI. Since the Ni–Co oxide film derived from Bath 1 exhibited a stronger magneto-electric effect, subsequent characterization was focused on this sample.

### Structural and MI characterization of the outperforming sample

#### Study of nanoscale defects size and distribution

VEPALS was conducted on the metallic Ni–Co film derived from Bath 1 (as-deposited), on the same film after annealing (as-annealed), and after annealing followed by gating at −20 V for 30 min (gated). This technique enables analyzing the defect-size-dependent positron annihilation lifetime of a given sample as a function of depth. The main goal was to observe the deconvoluted effects of oxidation and voltage treatment on the size and amount of small vacancy clusters (*τ*_1_, *i.e.* shortest lifetime component), grain boundaries or small pores (*τ*_2_, *i.e.* intermediate lifetime component) and voids (*τ*_3_, *i.e.* longest lifetime component). It should be noted that the mesopores as seen by FE-SEM were not observed by positrons, likely because of their openness to the surface, which allows for the *ortho*-Positronium escape to vacuum, hence, effectively hindering its annihilation in mesopores; therefore, the terms ‘small pores’ or ‘voids’ used here do not refer to mesopores but to smaller micropores, which still could be in the vicinity of the former.

**Fig. 2:**
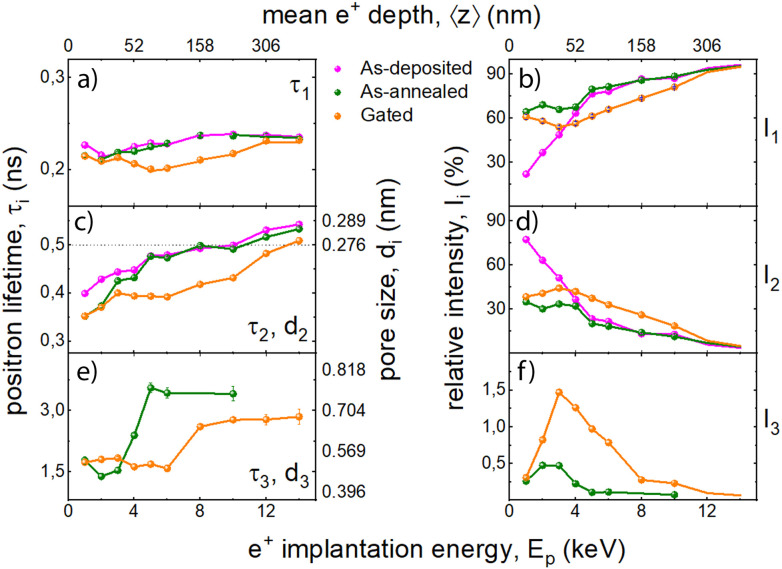
(a, c and e) Positron lifetime components *vs.* the mean penetration depth (*i.e.*, implantation energy, *E*_p_) for the different magnetic states of the film obtained from Bath 1 (as-deposited), the same film subject to annealing in air (as-annealed), and the annealed film voltage-treated at −20 V for 30 min (gated). (b, d and f) Symbolize the relative intensities of each of the defect size ranges, for the magnetic states studied. For lifetimes larger than 500 ps *ortho*-positronium annihilation contributions in micropores is expected. The respective lifetimes were recalculated to the pore sizes and given as the right *Y*-axis.^38^

Fig. 2 exhibits the depth-resolved lifetime components (*τ*_1_, *τ*_2_ and *τ*_3_) and their relative intensities, for the as-deposited, as-annealed and voltage-actuated samples. Since the sum of intensities of the *τ*_1_ and *τ*_2_ contributions is around 100% for any of the case studies, it can be assumed that most of the encountered defects are essentially vacancy clusters of different sizes. For a representative defect density at a mean depth of 52 nm (using 4 keV), the as-deposited sample shows values for *I*_1_ and *I*_2_ of 64% and 35%, respectively, with an almost negligible contribution from *I*_3_ (Fig. 2b–f). This indicates that small vacancy clusters are indeed the majority defect type.

Regarding the transition from the as-deposited to the as-annealed states, the thermal treatment causes an increase in the number of smaller vacancy clusters (associated with *τ*_1_) in the sub-surface region, while the number of larger vacancy clusters (linked to *τ*_2_) decreases there. At the same time the size of larger vacancy clusters (*τ*_2_) decreases slightly in the sub-surface region. The deeper regions seem not to be affected regarding the variation of vacancy clusters microstructure. It is important to interpret the relative intensity trends carefully, as the first few data points are noticeably skewed due to surface roughness and broken symmetry at the topmost layer of the films. In summary, annealing reduces the number of larger vacancy clusters as they possibly collapse, while the amount of small vacancy agglomerates increase (Fig. 2b and d). Essentially, annealing results in a significant decrease in pore size, as experimentally observed by FESEM (*cf.*Fig. 1a and b), however here VEPALS gives insights into the scale range not accessible with electron microscopy. In addition, annealing generates a small amount of microporosity (*τ*_3_), which is however only detectable in the sub-surface region, as the intensity of that lifetime component (*I*_3_) decreases to nearly zero after the depth of 50 nm.

Although Fig. 1 suggests that mesopores are mainly responsible for the enhanced MI, the contribution of micropores should not be ruled out. In principle, mesoporous materials made by electrodeposition are primarily mesoporous, and any microporosity is typically minor or incidental, not a defining feature. However, the results suggest that annealing promotes an increase in the population of smaller vacancy clusters (*I*_1_ associated with *τ*_1_) in the subsurface region, thereby imparting hierarchical porosity (micro- and mesoporous) to the film, which may collectively contribute to enhance the MI.

Interestingly, the transition from the as-annealed to the gated states induces the opposite effect: an increase in the number of larger vacancy agglomerations (*τ*_2_ and *I*_2_) and micropores (*τ*_3_ and *I*_3_) at the expense of smaller vacancy clusters (*τ*_1_). However, all three defect sizes are reduced. Regarding the relative variation of larger defects and voids (associated with *τ*_2_ and *τ*_3_) between these two states, the electric field induces a reduction in both defect sizes, even though the trend of increasing micropores density at the expense of smaller vacancy clusters remains the same. Considering that an oxygen vacancy can be regarded as a neutral or cationic defect only as a complex with a negatively charged vacancy, this could be attributed to the decreased oxygen vacancies, resulting from O^2−^ ion migration caused by the applied voltage, which leads to electrochemical reduction.

#### MI characterization

**Fig. 3:**
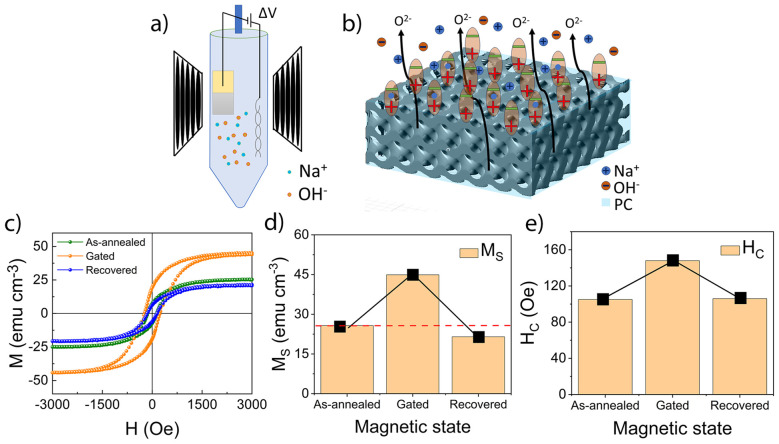
(a) Custom-built cell setup for liquid gating of the mesoporous Ni–Co oxide coating prepared on top of the Si/Ti/Au substrate. (b) Schematic representation of the mesoporous structure of the Ni–Co oxide layer, illustrating the charge arrangement for a negatively biased sample. (c) Room-temperature magnetic hysteresis loops for as-annealed (green), gated (orange) and recovered (blue) states; (d) and (e) are the corresponding *M*_S_ and *H*_C_ values measured at each state.

The mesoporous Ni–Co oxide film (obtained from Bath 1) shown in Fig. 1d underwent a more in-depth MI characterization. A scheme of the cell setup used for the *in situ* magneto-electric measurements and the material's mesoporous network are depicted in Fig. 3a and b, respectively. Note that the Si/Ti/Au/Ni–Co oxide sample was immersed in PC, with electrical contact established through the uncoated portion of the Au seed layer. Fig. 3b illustrates the formation of the EDL upon negatively polarizing the sample, where the PC dipoles align with their negative charge facing away from the surface, and the Na^+^ cations accumulate near the Ni–Co oxide surface.

Fig. 3c shows the hysteresis loops of the mesoporous Si/Ti/Au/Ni–Co oxide sample in the as-annealed state (green) and after biasing at −20 V for 30 min (green curve, labelled ‘gated’) and at +5 V for 90 min (blue curve, labelled as ‘recovered’). It is conjectured that O^2−^ ion migration from the Ni–Co oxide layer towards the electrolyte takes place upon negative biasing, which accounts for the increase in the magnetic moment at saturation from approximately 25 to 46 emu cm^−3^ (84% increase). Simultaneously, an increase of approximately 41% in *H*_C_ with respect to its initial value (‘as-annealed’ sample) is observed upon negative biasing. When the sample is biased at +5 V for 90 min, the magnetic moment at saturation decreases due to oxygen reincorporation in the Ni–Co oxide layer. Interestingly, its value remains lower than that of the initial ‘as-annealed’ state, which may seem counterintuitive. However, a detailed structural explanation is provided below to clarify this effect.

#### XRD and XPS characterization

**Fig. 4:**
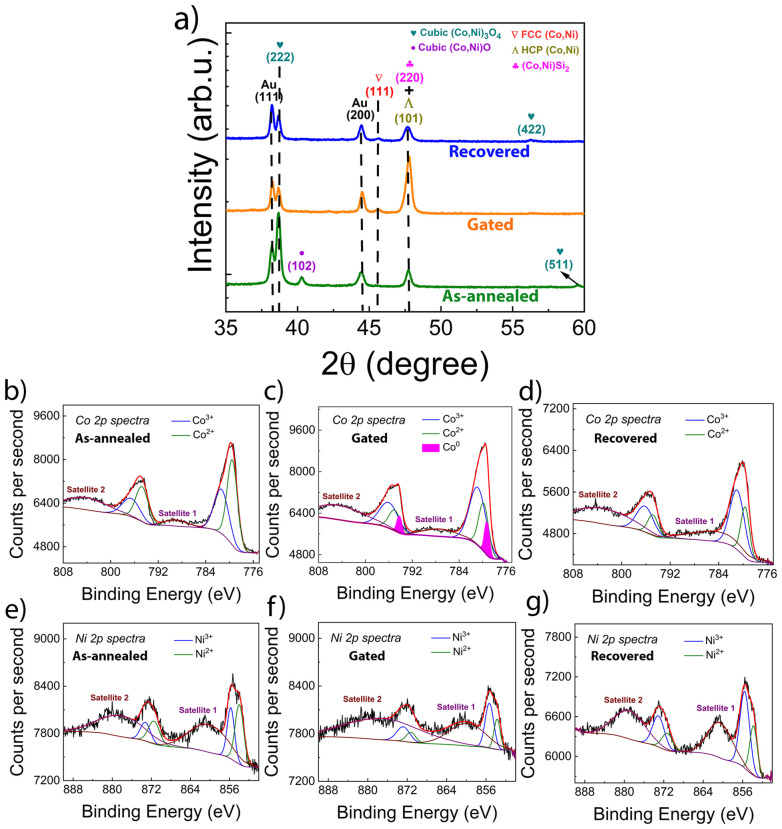
XRD patterns of the as-annealed, gated and recovered mesoporous Ni–Co oxide-coated Si/Ti/Au samples. Corresponding (b–d) Co 2p and (e and f) Ni 2p core-level XPS spectra taken from the utmost Ni–Co oxide layer.

The crystallographic structure of the mesoporous Ni–Co oxide film was studied by XRD. Fig. 4 shows the XRD *θ*–2*θ* patterns for the as-annealed, gated and recovered states. The as-annealed sample showed peaks compatible with (Co,Ni)_3_O_4_ (mp-18748), Co(Ni)O (mp-19079), FCC (Co,Ni) and (Co,Ni)Si_2_ phases, besides a few reflections originating from the Au seed layer. While the occurrence of oxide phases ((Co,Ni)_3_O_4_ and Co(Ni)O) was expected due to heat treatment in air, the presence of the metal silicide phase ((Co,Ni)Si_2_) at 2*θ* = 47.7° suggests diffusion of silicon from the substrate into the Ni–Co layer occurred during annealing. This phenomenon has been previously observed, even in the presence of a titanium buffer layer, suggesting that the latter is not an efficient barrier against diffusion of Si.^39–41^ Upon annealing at 450 °C, Ti forms titanium silicide (TiSi_*x*_),^42^ which consumes Si and Ti, so instead of blocking diffusion, the layer is being chemically transformed. Once silicide starts forming, the structure becomes even more permeable. At ≈363 °C, it has been reported that Au and Si form a eutectic liquid phase^43^ which dramatically accelerates Si migration upward and Au penetration downward. So, Au can enhance silicon migration rather than prevent it. The weak reflection at 2*θ* = 45.6° is ascribed to FCC (Co,Ni), and accounts for the remanent ferromagnetic signature evidenced in the hysteresis loop recorded for this sample, since complete oxidation of the Ni–Co layer during the annealing was not achieved. Considering the bath formulation, synthetic conditions, and chemical composition of the electroplated layer (Ni_55_Co_45_), the FCC phase is expected.^44^

Upon liquid-gating the sample at −20 V for 30 min, the peaks corresponding to (Co,Ni)_3_O_4_ drastically decrease in intensity, while the peak associated with Co(Ni)O even disappears. Simultaneously, the intensity of the peak located at 2*θ* = 47.7° increases, which might be explained by changes in the texture and crystallinity of the paramagnetic (Co,Ni)Si_2_ phase.^45^ On the other hand, the position of this peak coincides with that of HCP (Co,Ni) phase. Therefore, a minor contribution from ferromagnetic HCP (Co,Ni) cannot be ruled out, suggesting the possible formation of HCP (Co–Ni) clusters. Further, the peak at 2*θ* = 45.6° ascribed to metallic FCC (Co,Ni) also increases in intensity. These results strongly suggest that O^2−^ ions have indeed diffused toward the material/electrolyte interface, leaving behind ferromagnetic metallic clusters within the Ni–Co oxide/silicide layer. The subsequent application of +5 V for 90 min is supposed to bring oxygen anions back into the material. This results in a relative increase in the intensity of the (Co,Ni)_3_O_4_ phase and a decrease in the intensity of the peaks corresponding to the metallic phases (*i.e.*, HCP and FCC Ni–Co). Note, however, that the XRD pattern of the ‘recovered’ state differs from that of the ‘as-annealed’ state, suggesting that oxygen reinsertion into the material occurs in chemical environments different from those it originally occupied. This might be the reason for the dissimilar *M*_S_ values between as-annealed and recovered states.

Aiming to study the chemical environment of the Ni and Co atoms in the mesoporous Ni–Co oxide layer at the different states, XPS measurements were carried out. The core level spectra of Co 2p and Ni 2p for the as-annealed sample consists of a combination of both Co and Ni with both +2 and +3 valence states, as proven by the fitted peaks shown in Fig. 4b and e, respectively. The binding energy of 779.3 eV for Co 2p_3/2_ can be assigned to Co^2+^ oxidation state according to the literature.^46,47^ Similarly, the Ni 2p signal could be deconvoluted considering Ni^2+^ and Ni^3+^ contributions. The binding energy of 854.0 eV for Ni 2p_3/2_ matches Ni^2+^ oxidation state.^48,49^ Although magnetic measurements indicated that this sample is weakly magnetic, the absence of metallic Co and/or Ni signals in the XPS analysis is attributed to the surface sensitivity of the technique. On the other hand, the XPS pattern of the as-gated film (−20 V for 30 min) consists of an additional Co^0^ peak along with the +2 and +3 peaks, as shown in Fig. 4c, confirming the diffusion of O^2−^ ions from the Ni–Co oxide layer towards the liquid electrolyte. Meanwhile, the Ni 2p spectra (Fig. 4f) did not show the contribution of zero-valent Ni, suggesting the electric field-assisted migration of oxygen anions bonded to Ni is less favorable than that of oxygen anions bonded to Co, as previously reported.^35^

After successive positive voltage treatment with +5 V for 90 min, the 2p spectra of both Co and Ni show again the +2 and +3 oxidation state-related peaks (Fig. 4d and g), without any hint of metallic Co (Fig. 4d). Nonetheless, the +2 contribution decreased significantly in the recovered state, as compared to the as-annealed state, in favor of the +3 oxidation state. Additionally, as a common feature for all the studied spectra, shake-up satellite peaks located at 786 eV and 803 eV (in the Co 2p spectra)^50^ and at 861 eV and 882 eV (in the Ni 2p spectra),^51^ appear as a consequence of the degenerated magnetic spin states. These are associated to sudden external shell excitations, which arise from the agitation of the central potential after photoionization of an inner electron.^52^

Overall, the XPS data match the findings of XRD, for which the presence of metallic HCP and FCC Ni–Co was verified in the treated sample. Also, the fitting of the XPS patterns indicate that Co^2+^/Co^3+^ ratio decreases from the as-annealed to the recovered states, which suggests a complex oxygen entryway phenomenon into the host mesoporous structure upon positive biasing. Note that the (102) peak present in the as-annealed sample and ascribed to (Co,Ni)O is nearly absent in the pattern for the recovered sample (Fig. 4a).

#### Effect of cycling frequency on the film's endurance

**Fig. 5:**
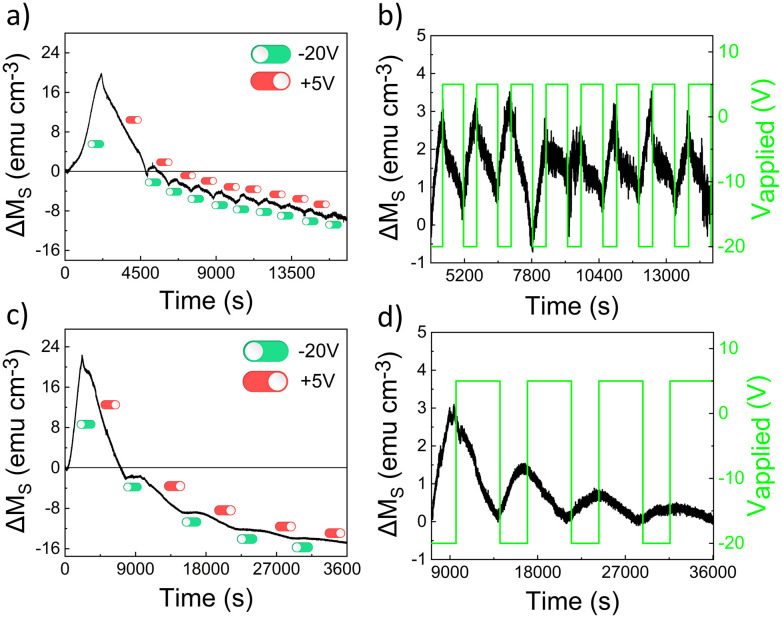
Time-evolution of Δ*M*_S_ for (a) high and (c) low frequency cycling of the mesoporous Ni–Co-coated Si/Ti/Au sample. (b and d) A negative-exponential corrected baseline is used to highlight the amplitude of cycles from the second onward.

With the aim to clarify whether reversible phase transformations took place upon voltage application to the sample, a series of magneto-ionic cyclability tests were carried out, subjecting the mesoporous Ni–Co-coated Si/Ti/Au sample to two different frequency conditions, namely negative/positive DC biasing for 500 s/800 s (high frequency) and 1800 s/5400 s (low frequency). Fig. 5a and c show that the variation in saturation magnetization (Δ*M*_S_) and the amplitude of the cycles decrease over time. In fact, a significant decrease in amplitude occurs from the 1^st^ to the 2^nd^ cycle, after which the differences become much smaller. This is more clearly seen in Fig. 5b and d, which show the second and subsequent cycles after baseline removal using a decaying exponential function. Note also that the values for Δ*M*_S_ are negative from the 2^nd^ cycle onward, as the starting *M*_S_ value for each cycle is lower than that of the previous one. This in agreement with the lower *M*_S_ of the recovered sample compared to the as-annealed one in Fig. 3c, and the effect continues steadily with increasing number of cycles. On the other hand, cyclability performance depends on frequency: at higher frequencies, the sample withstands more cycles before becoming unresponsive. Specifically, while only five cycles can be run at low frequency, around ten cycles are achievable at higher frequency (*cf.*Fig. 5a and c). The low-frequency cyclability suggests the occurrence of irreversible microstructural and/or compositional changes in the sample. Fig. S5 of the SI reinforces the hypothesis that the sample cycled at low frequency underwent microstructural and/or compositional irreversible changes, possibly during positive biasing (+5 V, 5400 s). This is evidenced by the absence of saturation magnetization modulation, even after further gating at a relatively large voltage of −100 V for 1 h.

#### STEM-EDX & HRTEM characterization

**Fig. 6:**
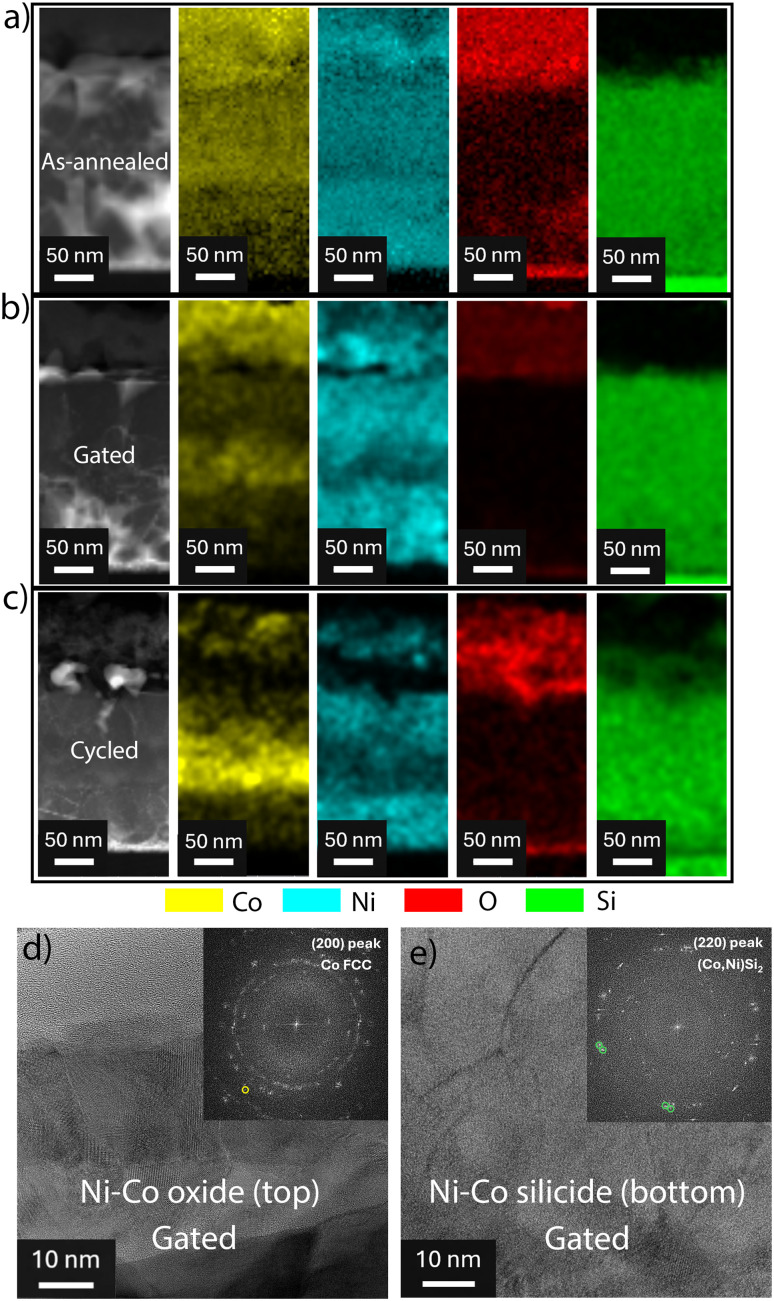
HAADF-STEM image (left) and corresponding EDX mapping for (a) as-annealed, (b) gated and (c) low-frequency cycled Si/Ti/Au/Ni–Co oxide sample. HRTEM and corresponding FFT (inset) from the top (d) and bottom (e) regions of the sample in the gated state. The spots enclosed in the yellow circles in (d) have interplanar distances compatible with (200) FCC-Co, while the green circles in (e) enclose spots whose interplanar distances match the (220) (Co,Ni)Si_2_ phase.

Cross-section lamellae of the mesoporous Si/Ti/Au/Ni–Co oxide sample in as-annealed and gated states were prepared by FIB for subsequent characterization by means of STEM, EDX and HRTEM. A third lamella corresponding to the sample at the end of the low-frequency cycling process in Fig. 5c was also examined to better understand the restructuring of the Ni–Co oxide/silicide layer upon cycling. Fig. 6a shows that, already in the as-annealed state, the distribution of Ni, Co and O elements within the sample is not homogeneous. In fact, Co is rather accumulated towards the top surface of the film where oxygen is mostly concentrated. A characteristic step of the relative intensity from the positron analysis (Fig. 2b and d) results from that aggregation. Co segregation towards the top surface has been previously observed in electrodeposited, dense Ni–Co disks subjected to annealing in air.^35^ Ni is more homogeneously distributed although it is a bit more concentrated at the bottom region of the film (*i.e.*, close to the silicon substrate). The diffusion of silicon into the Ni–Co layer is also evident in the EDX mapping. Interestingly, silicon has diffused up to the top Ni–Co oxide region, *i.e.*, roughly 100 nm below the film surface. Note that the nickel and/or cobalt silicide phase is not ferromagnetic,^45,53^ which explains why the sample does not show higher *M*_S_ even though only the top region of the Ni–Co electrodeposited layer was effectively oxidized during annealing.

The gated sample (*i.e.*, the one treated at −20 V for 30 min) shows, one the one hand, that the oxygen content has severely decreased (see the top part of the sample), thereby proving its migration towards the electrolyte. On the other hand, the silicon signal remains comparable. Importantly, the distribution of Ni and Co has changed with respect to the situation in the as-annealed sample. Ni and Co exhibit an uneven, band-like distribution. Co accumulates more at the top region of the film, where oxygen is heavily depleted, likely enhancing the sample's ferromagnetic signal. Ni is more homogeneously distributed in depth but still forms bands. Finally, the sample which underwent 5 voltage cycles (*cf.*Fig. 5c) clearly shows again oxygen in the top region of the film and the band-like distribution of Co and Ni. Again, the top oxide layer and the silicide layer beneath can be clearly distinguished. The STEM-EDX mappings indicate that, in fact, only the top 100 nm and not the whole Ni–Co-based layer is contributing to the observed MI effects. In other words, the silicide phase functions as working electrode, inducing the occurrence of the MI phenomenon exclusively in the top region of the sample. The magnetizations in Fig. 1 were normalized to the whole coating thickness (*i.e.*, ≈350 nm). On the other hand, comparing the as-annealed and cycled samples (*cf.*Fig. 6a and c) reveals significant microstructural and compositional changes, which are likely responsible for the sample's limited endurance.

Fig. 6d and e show representative HRTEM images of the top and bottom parts of the gated sample (*i.e.*, where the Ni–Co oxide and silicide phases are located, respectively). These HRTEM images prove the polycrystalline nature of the sample, as expected. A few small voids can be observed in Fig. 6d that may correspond to the mesopores. The Fast Fourier Transform of the top region (inset in Fig. 6d) shows, in addition to spots compatible with oxide phases, discrete spots matching (200) FCC (Co,Ni) phase, enclosed in yellow circles (*d* = 1.772 Å). This agrees with the XRD and XPS data for this sample, confirming that negative biasing drives the migration of oxygen ions out of the sample, leaving behind ferromagnetic clusters. Meanwhile, the bottom region of the same sample shows spots enclosed in green circles whose interplanar spacing (*d* = 1.894 Å) match the (220) (Co,Ni)Si_2_ phase, in agreement with the formation of the Ni–Co silicide phase during annealing.

To elucidate the structural modifications of the Ni–Co oxide-coated Si/Ti/Au sample upon cycling, XRD patterns were collected after 1 and 5 low-frequency cycles. A marked decrease in the intensity of the peak attributed to the (Co,Ni)_3_O_4_ phase was observed, whereas the (Co,Ni)Si_2_ peak remained largely unchanged (see Fig. S6). This behavior suggests the occurrence of severe structural changes in the active Co–Ni oxide layer, such as amorphization, which may hinder O^2−^ migration.

Our results reveal how voltage-driven oxygen diffusion in porous Ni–Co oxide can result in significant changes in magnetic properties. The approach might be extrapolatable to other families of materials (*e.g.*, rare-earth nickelates), where incorporation of oxygen ions *via* oxygen annealing procedures has been recently reported to induce remarkable changes in the resistive switching behavior.^54^

## Conclusions

To the best of our knowledge, this work represents the first instance of a mesoporous binary metal oxide being investigated under electrolyte-gating MI. While nearly dense metal oxides (typically produced by physical methods) and mesoporous alloy structures obtained *via* electrodeposition have previously been studied in this context, no material combining both characteristics has been reported to date. Our results prove that the increase in the surface-to-volume ratio of Ni–Co oxide films favor liquid electrolyte gating, leading to enhanced modulation of their *M*_S_ upon negative/positive biasing. Under negative polarization, an increase in *M*_S_ of up to 84% has been observed in 350 nm thick mesoporous films grown by micelle-assisted electrodeposition (55 at% Ni) and subsequently annealed in air, largely exceeding the relative changes observed in less porous films with similar composition and thickness (8–25%). Remarkably, the *M*_S_ measured after applying positive bias is lower than its initial value, suggesting that oxygen migration in and out of the material follows a complex pattern. Annealing the as-deposited mesoporous Ni_55_Co_45_ film in air at 450 °C has, on the one hand, facilitated oxidation of the top layer (enabling oxygen diffusion toward the electrolyte during subsequent negative biasing) and, on the other hand, largely preserved the mesoporosity. However, silicon from the substrate inevitably diffuses into the bottom part of the Ni–Co film. The formation of the silicide phase, which is non-ferromagnetic but electrically conductive, reduced the effective thickness of the MI target (the Ni–Co oxide film) from 350 nm to approximately 100 nm, resulting in an improved MI response.

## Author contributions

A. Arrendondo-López: investigation, validation, formal analysis, data curation, writing – original draft, visualization. K. Eiler: methodology, validation, formal analysis, writing – review & editing. A. Quintana: formal analysis, writing – review & editing. Z. Ma, E. Hirschmann, A. Wagner: investigation. M. O. Liedke: investigation, writing – review & editing. E. Menéndez: conceptualization, methodology, writing – review & editing, supervision, project administration. J. Sort: conceptualization, methodology, writing – review & editing, resources, supervision, project administration, funding acquisition. E. Pellicer: conceptualization, methodology, writing – original draft, resources, supervision, project administration, funding acquisition.

## Conflicts of interest

There are no conflicts to declare.

## Supplementary Material

NR-018-D6NR00524A-s001

## Data Availability

Data for this article is made available at https://dataverse.csuc.cat/ Supplementary information (SI): *E*–*t* curves recorded during Ni-Co deposition, representative EDX, SEM images after annealing, ΔM *vs*. time curves, XRD patterns of cycled samples. See DOI: https://doi.org/10.1039/d6nr00524a.
