# Supplementary material for: Voltage control of magnetism in Ni–Co oxide mesoporous films: impact of porosity on oxygen magneto-ionics performance
Source: Nanoscale. 2026 Apr 22;18(20):10724–35. doi: 10.1039/d6nr00524a (PMC13100708; doi:10.1039/d6nr00524a)
Supplement: NR-018-D6NR00524A-s001 [file NR-018-D6NR00524A-s001.pdf]

Supplementary Information (SI) for Nanoscale.
This journal is © The Royal Society of Chemistry 2026

Supplementary Information

Voltage Control of Magnetism in Ni-Co Oxide Mesoporous Films: Impact of Porosity on Oxygen Magneto-Ionics Performance

Aitor Arredondo-López[a], Konrad Eiler[a], Alberto Quintana[a,b], Zheng Ma[a], Maciej Oskar Liedke[c], Eric Hirschmann[c], Andreas Wagner[c], Enric Menéndez[a,], Jordi Sort[a,b,d*], Eva Pellicer[a,*]*

[a] *Departament de Física, Universitat Autònoma de Barcelona, E-08193 Cerdanyola del Vallès, Spain*

[b] *Catalan Institute of Nanoscience and Nanotechnology (ICN2), CSIC and BIST, 08193 Barcelona, Spain*

[c] *Institute of Radiation Physics, Helmholtz-Zentrum Dresden – Rossendorf, Dresden 01328, Germany*

[d] *Institució Catalana de Recerca i Estudis Avançats (ICREA), Pg. Lluís Companys 23, E-08010 Barcelona, Spain*

Corresponding authors, E-mail: enric.menendez@uab.cat, jordi.sort@uab.cat, eva.pellicer@uab.cat

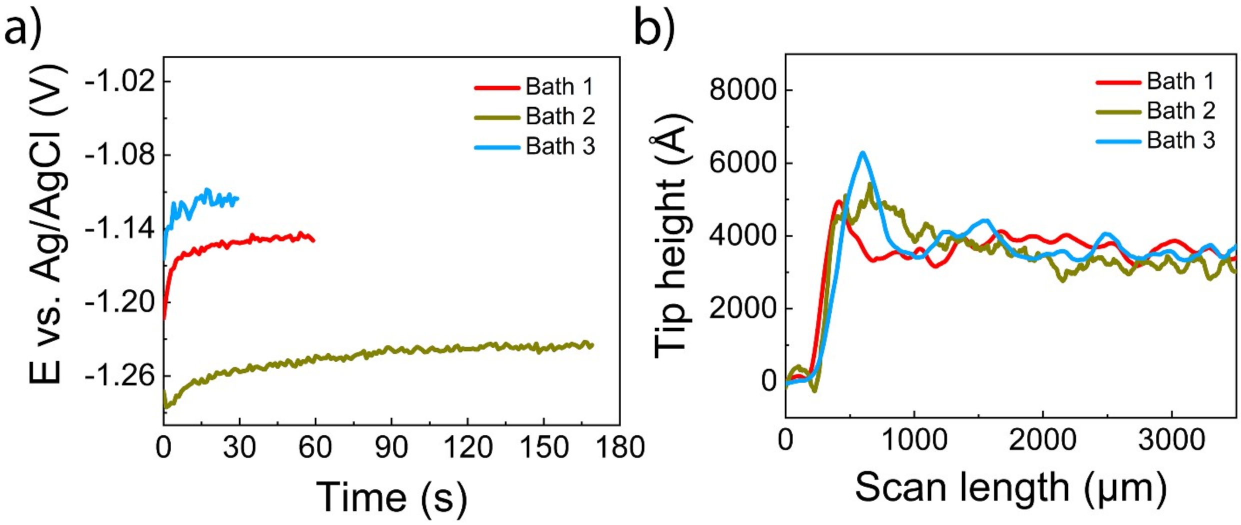

Figure S1. a) E-t curves recorded during the deposition of the Ni-Co films from the three different baths and b) corresponding film thickness measured as 'tip height' versus scan length by mechanical profilometry. The tip was placed on the step formed by the Ni-Co layer and the gold surface. The step profile was recorded as the tip moved deeper into the Ni-Co layer.

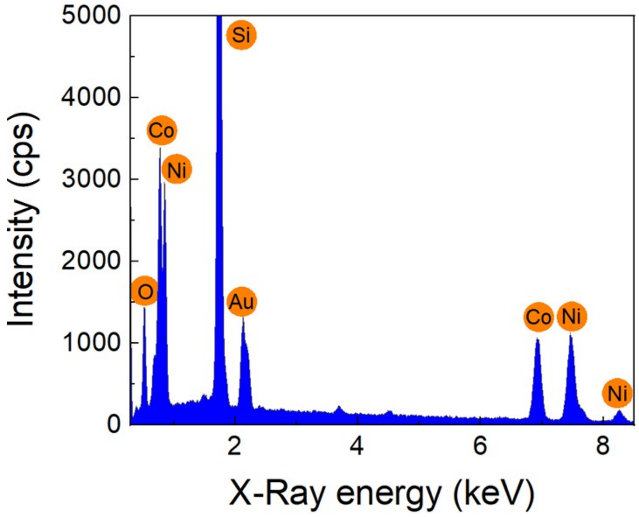

Figure S2. EDX pattern of the Ni-Co film obtained from Bath 1. The Si and Au signals belong to the substrate.

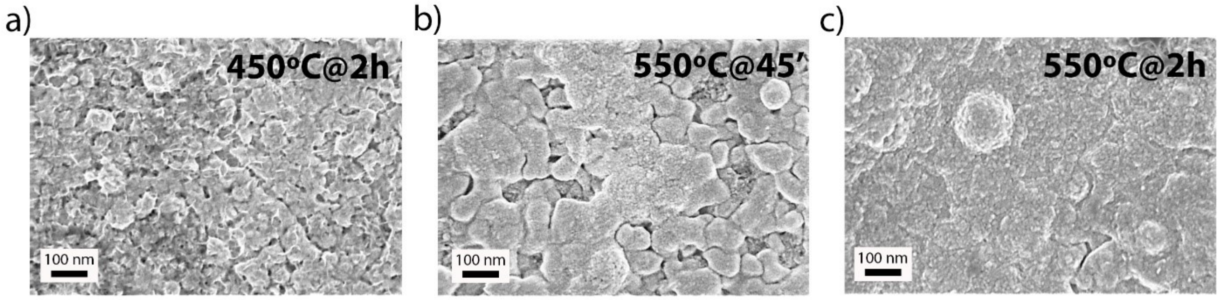

Figure S3. SEM images of the mesoporous Ni-Co film obtained from Bath 1 after annealing at a) 450 ºC for 2h, b) 550 ºC for 45 min, and c) 550 ºC for 2 h.

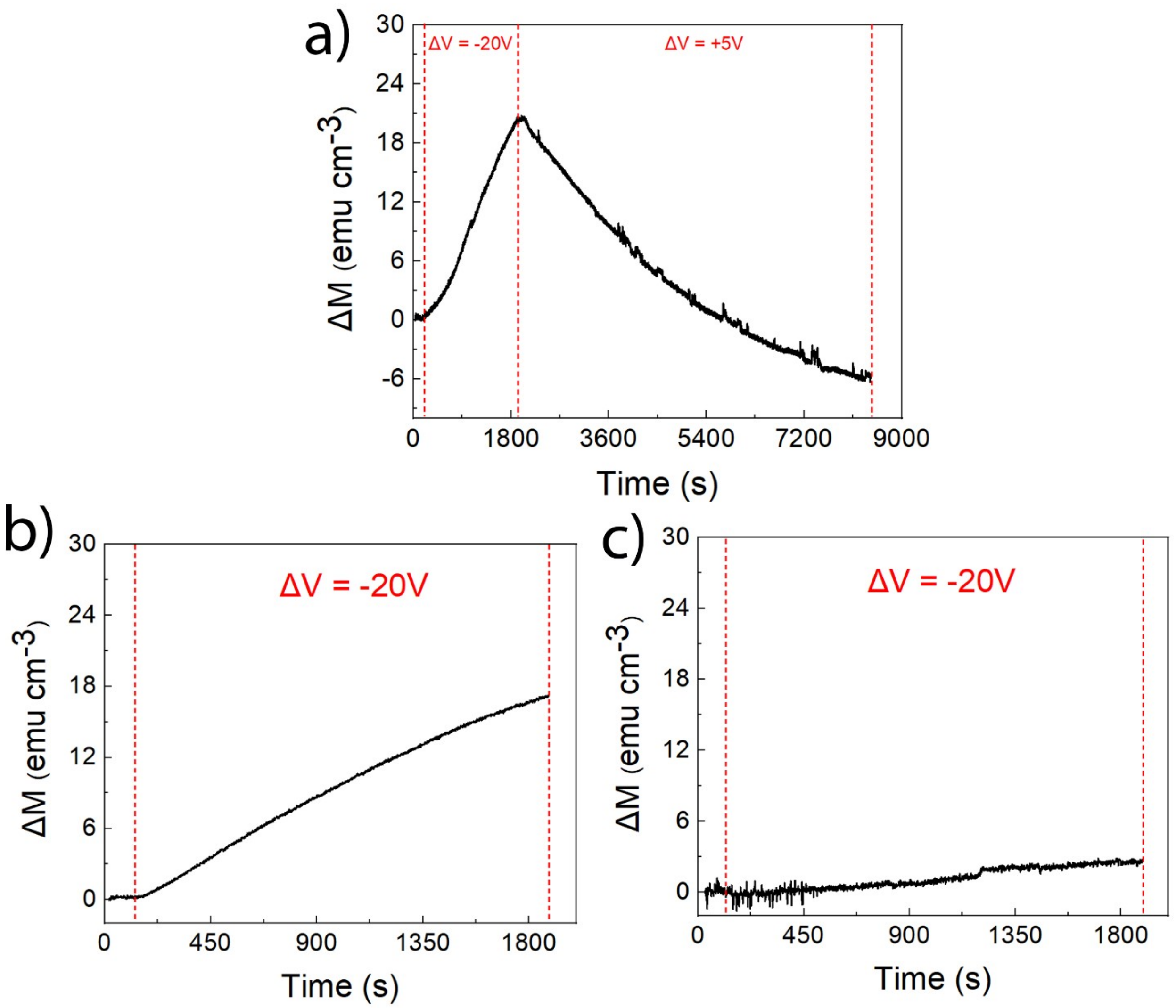

Figure S4. Evolution of Δ*M* vs time for the as-annealed Ni-Co films obtained from Baths 1 (a), 2 (b) and 3 (c) upon application of –20 V for 1800 s. The response recorded upon a subsequent positive biasing of the sample in [illegible] (a) is also shown.

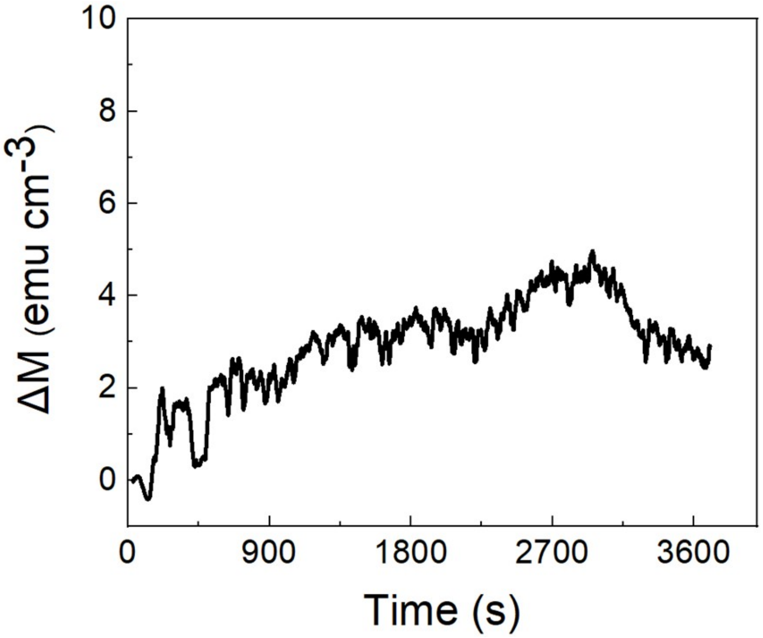

Figure S5. Evolution of ΔM with time upon electrolyte-gating the mesoporous Si/Ti/Au/Ni-Co oxide sample at –100V. The same sample had previously been subjected to 5 negative (–20 V, 800 s) / positive (+5 V, 5400 s) cycles (low frequency), suggesting the occurrence of irreversible microstructural and/or compositional changes.

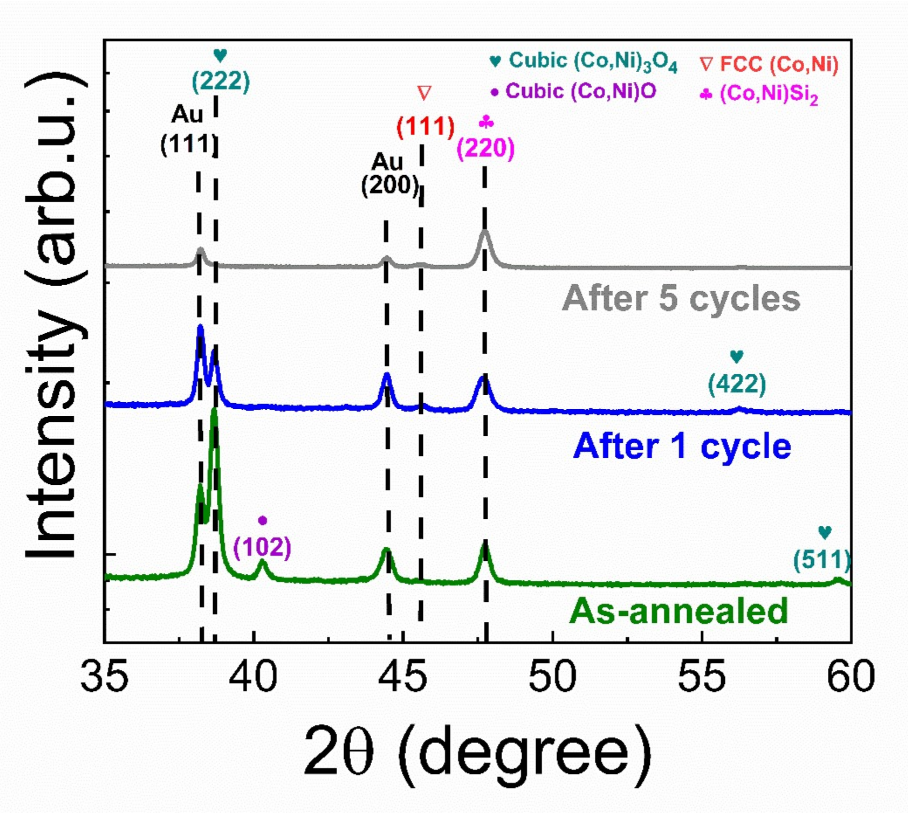

Figure S6. XRD patterns of the As-annealed, 'After 1 cycle' (corresponding to the 'Recovery' state in Figure 4(a) of the main manuscript), and 'After 5 cycles' for mesoporous Ni-Co oxide-coated Si/Ti/Au samples. Negative and positive biasing correspond to –20 V for 30 min and +5 V for 90 min per cycle, respectively.
